# Feto-maternal outcomes and Glycemic control in Metformin versus insulin treated Gestational Diabetics

**DOI:** 10.12669/pjms.335.13286

**Published:** 2017

**Authors:** Rabia Arshad, Samia Khanam, Fuad Shaikh, Nasim Karim

**Affiliations:** 1 Dr. Rabia Arshad, MBBS, M. Phil, Assistant Professor, Department of Pharmacology, Altamash Institute of Dental Medicines, Karachi, Pakistan; 2 Dr. Samia Khanam, MBBS, M. Phil, Assistant Professor and Head, Department of Pathology, Altamash Institute of Dental Medicines, Karachi, Pakistan; 3 Dr. Fuad Shaikh, MBBS, M.Phil, Associate Professor, Department of Pharmacology, Dow University of Health Sciences, Karachi, Pakistan; 4 Dr. Nasim Karim, MBBS, M. Phil, Ph D, Post Doc (USA), Head of Pharmacology Department, Bahria University Medical and Dental College, Sailors Street, Adjacent PNS Shifa, Defence Phase-II, Karachi, Pakistan

**Keywords:** Gestational diabetes, Insulin, Fetal outcomes, Maternal outcomes, Metformin

## Abstract

**Objective:**

To evaluate and compare feto-maternal outcomes and glycemic control in metformin versus insulin treated gestational diabetics.

**Methods:**

The study was conducted in 2010- 2012 as a part of M. Phil at Civil hospital, Lyari General Hospital and Mamji Hospital in Karachi. After written informed consent, 71 GDM diagnosed females with WHO criteria were enrolled. They were divided into two groups. Group-A, 32 females were given oral metformin 500 mg TDS while Group-B, 39 females were given insulin 0.8-0.9 mg/kg/day in two divided doses subcutaneously. Patients were followed till term. Feto-maternal outcomes were evaluated in 25 patients in each group who completed the study.

**Results:**

When groups were compared, newborns in Group-B were significantly more in weight (p=0.01). Significant numbers of babies were delivered after 38 weeks of pregnancy in Group-B (P=0.021). There were two intrauterine deaths and significantly higher HbA_1_C at term in Group-B. (P=0.03). FBS at term was non-significant (p=0.079) and there was more number of cesarean sections due to feto-maternal disproportion in Group-B (28% vs.2%). Results analyzed for glycemic control before and after the treatment revealed that FBS was statistically less in Group-A (p=0.00) whereas for Group-B the value of FBS and HbA_1_C was statistically high. (p=0.002 & 0.04 respectively).

**Conclusion:**

Metformin has produced better effects on feto-maternal outcomes and glycemic control in comparison to Insulin in GDM.

## INTRODUCTION

Glucose intolerance occurring during the prenatal period, Gestational Diabetes Mellitus (GDM), is usually identified in the second trimester of pregnancy. The causes of pancreatic beta - cell dysfunction in pregnancy, leading to insulin deficiency in GDM, are not fully understood. Main factors are adiposity in pregnancy, autoimmune beta cell dysfunction, decreased insulin secretion and insulin resistance.^1^ Pathophysiology is strongly suggestive of circulating placental and maternal hormones leading to a decrease in glucose sensitivity by the tissue receptors and subsequent increases in the carbohydrate tolerance.

From 1995 to 2005, gestational diabetes had amplified by 45% overall, with the world wide prevalence from 3.0 to 4.4%.^2^ Women of South Asia are seems to be at the utmost risk for GDM with the prevalence rate being 3.2-3.5%. GDM is almost 72%more frequently seen in patient’s age ≥ 30 years, 50% more common with positive family history and 29% occur more in those with raised body mass index.^3^

Traditionally Insulin has been known as an effective therapy for GDM as it shows adequate control of raised blood glucose levels.^4^ It was initially prepared as a lifesaving drug for Type-I insulin deficient diabetic patients but later was also used for gestational diabetes as most of the oral medications were considered to be teratogenic. For decades it has been used for the treatment of diabetes in pregnancy.^5^ It requires proper storage and is associated with high incidence of maternal morbidity and mortality such as maternal hypoglycemia, macrosomic babies leading to assisted or surgical deliveries and more weight gain in pregnancy.^6^

Metformin, an oral anti diabetic drug is being used since 1960 in patients with Type-2 diabetes mellitus. It has now been upgraded to a Category B drug as it is not associated with any fetal congenital anomalies.^7^ It was initially used to reduce insulin resistance in females with the polycystic ovarian syndrome and as it showed remarkable results with no side effects in pregnancy so was considered a better alternative for the management of GDM.^8^ It can be a logical treatment for pregnant females as it produces the euglycemic state by improving insulin sensitivity, reducing hepatic gluconeogenesis and increasing peripheral glucose uptake and utilization.^9^ Present study was designed to evaluate and compare the effects of metformin and insulin on feto-maternal outcomes and glycemic control in GDM patients.

## METHODS

This Karachi based study was started in Lyari General Hospital, Mamji Hospital and Civil Hospital, after obtaining approval from Institutional Review Board and Ethical Committee of Dow University, Karachi. It was conducted during 2010-2012 as a part of M.Phil. For this study, high risk patients were recruited from hospitals after obtaining a written informed consent. High risk patients include those with bad obstetric history (previous abortions, intra uterine deaths or still births), strong family history, obese patients and patients with history of gestational diabetes in previous pregnancies. Seventy one diabetic pregnant females were enrolled in the study in second trimester after confirmation for GDM with Oral Glucose Challenge Test and Oral Glucose Tolerance Test, according to WHO criteria. These were divided randomly into two groups. The odd numbers patients (GROUP A=32 patients) were given Tab. Metformin, 500mg thrice daily, with dose escalation therapy prescribed in once daily dose in morning and then increased to thrice daily dose as required, depending upon the glycemic control of the patients. Even number (GROUP B=39 patients) received subcutaneous insulin. A total Insulin dose was calculated using 0.8 units /kg /day in 2^nd^ trimester and 0.9 units /kg/day in 3^rd^ trimester. 2/3 of the dose was prescribed in the morning and 1/3 of the dose was advised in the night. They were counseled well for strict diet control with provided diet charts (1800-2000 kcal /day) and were also advised for 30 minutes of walk at least thrice weekly. These patients were followed fortnightly up till 32 weeks and weekly till term. Maternal outcomes were then re-evaluated at 36 weeks of pregnancy with fetal outcomes and mode of delivery, being noted soon after the child birth.^10^ Data was subjected to statistical analysis and results were tabulated using SPSS-16. Chi square, independent T-test and Fisher exact tests were applied accordingly and p-value of 0.05 or more was considered significant.

## RESULTS

Results were compiled for 50 patients who completed the study with 25 patients in each group. By completion of study 21 patients had dropped out due to various reasons or delivered elsewhere and were excluded from the study.

When maternal characteristics were evaluated no difference was observed between age and weight of the patients in Group-A versus Group-B (p=0.09 & p>0.99 respectively). There were statistical differences present between the fasting blood sugar (FBS) and random blood sugar level (RBS) between the groups (p=0.039 & 0.001 respectively). When glycated hemoglobin (HbA_1_C) was compared statistically, results were non-significant (P=0.182) (Table-I).

**Table-I:** Baseline Maternal Characteristics Group-A v/s Group-B (n=50).

*S. No.*	*Characteristics*	*Group-A (n=25)*	*Group-B (n=25)*	*p-value*

		*Mean ± S.D*	*Mean ± S.D*	
1.	Age (years)	29.76±3.41	31.60±4.27	0.099
2.	Weight(kg)	77.90±7.65	77.9 ±9.03	NA
3.	FBS-1(mg/dl)	104.40±13.12	117.9 ±29.06	0.039*
4.	RBS(mg/dl)	171.16±37.44	239.16±69.78	<0.001*
5.	HbA_1_C--1 (%)	5.28±0.42	5.43±0.34	0.182

Group-A: Metformin with diet control and exercise, Group-B: Insulin with diet control and exercise

* Statistically significant (Independent T-test applied), FBS1: fasting blood sugar level at enrollment HbA_1_C-1: Glycated hemoglobin at enrollment.

When fetal outcomes were evaluated the babies of Group-B were significantly more in weight as compared to Group-A (p=0.01). Apgar score was statistically non-significant between the groups. On comparing delivery age, the results were statistically significant and more numbers of babies were delivered after 38 weeks of pregnancy in Group-B (P=0.021). All the babies were healthy and alive in Group-A whereas there were two intrauterine deaths at term in Group-B. When maternal outcomes were tabulated there were significant differences between Group-A and B for HbA_1_C at term, significantly higher in Group-B (P=0.03). FBS at term was non-significant between the groups (p=0.079). There were more number of cesarean sections in Group-B due to feto-maternal disproportional ratio as babies were bigger and heavier (28% vs.2%) (Table-II).

**Table-II:** Fetal and Maternal outcomes Group A v/s B (n=50).

*Fetal outcomes*	*Group-A (n=25)*	*Group-B (n=25)*	*P- value*

	Mean±SD	Mean±SD	
Weight of the baby (kg)	3.14±0.32	3.44±0.46	0.01*
Apgar score	8.64±0.56	8.22±2.43	0.39
	*n (%)*	*n (%)*	
*Delivery age*			0.021*
37 weeks	19(76%)	11(44%)	
38 weeks	6(24%)	14(56%)	
*Condition of baby*			0.49@
Alive baby	25(100%)	23(96%)	
Intrauterine death	0	2(8%)	
Still birth	0	0	
*Maternal outcomes*	Mean±SD	Mean±SD	
HbA_1_C-2 (%) (36weeks)	5.42±0.34	5.72±0.35	0.003*
FBS -2 (36 weeks)	93.48±11.9	102.08±20.63	0.079
	*n (%)*	*n (%)*	
*Mode of delivery*			NA
Normal vaginal	7(28%)	5(20%)	
Assisted delivery	2(8%)	3(12%)	
Cesarean section	16(64%)	17(68%)	
Cesarean due to bad obstetric history	14(56%)	10(40%)	
Cesarean due to Feto–maternal disproportion	2(8%)	7(28%)	

Group-A: Metformin with diet control and exercise, Group-B: Insulin with diet control and exercise, HbA_1_C-2: Glycated hemoglobin at 36 weeks, FBS-2: fasting blood sugar at 36 weeks,

* statistically significant result (chi square and independent T test applied), NA: chi-square test not applicable, @: Fisher exact test applied.

When results were analyzed for glycemic control before and after the treatment in the same groups it was observed that FBS were statistically less in Metformin treated females (p=0.00). HbA_I_C values were statistically non-significant before and after the treatment in Group-A. For Group-B the value of FBS was statistically high at term (p=0.002). Even HbA_1_C levels were statistically more at term in Group-B females (p= 0.04) (Table-III).

**Table-III:** HbA_1_C and FBS at enrollment and at 36-37 weeks of pregnancy Group A v/s B (n=50).

*Groups*	*At enrollment*	*At term*	*P- Value*

	Mean ± SD	Mean ± SD	
Group-A FBS	104.40±13.12	93.43±11.93	0.00*
Group-A Hba_1_c	5.284±0.42	5.420±0.346	0.061
Group-B FBS	117.92±29.06	102.08±20.64	0.002*
Group-B Hba_1_c	5.43±0.345	5.68±0.506	0.04*

Group-A: Metformin with diet control and exercise, Group-B: Insulin with diet control and exercise

HbA_1_C: Glycated hemoglobin; FBS: fasting blood sugar,

* statistically significant result (Paired T-test applied).

## DISCUSSION

Gestational diabetes mellitus is a growing health issue in many parts of the world. Our population is at higher risk to develop this disorder because of genetic, social, and environmental factors.^11^ Gestational diabetes has serious, long-term consequences for both baby and mother. Early diagnosis and management can surely improve the outcomes for these women and infants.^11^

Base line values for maternal age, maternal weight and HbA_1_C in both the groups showed no statistical difference though they have different FBS and RBS levels. Similar groups were taken by Balani for his research.^12^

When fetal outcome was evaluated it was observed that babies born to mothers on insulin treatment were significantly more in weight as compare to mother given oral metformin. (3.14 kg vs. 3.44, p=0.01). A randomized clinical trial conducted by Niromanesh concluded that babies born to mother given insulin were heavier than metformin treated GDMs.^13^ Recent study by Marques declared that there were no differences in fetal outcomes in metformin and insulin groups when compared together except neonatal hypoglycemia which was more in the insulin treated group.^14^ This study was done with much large sample size and a different study design could be the reason for non-similarity in the results of this study with ours. There was no difference for apgar score between both the groups in our study. Similar were the results documented by Rowan upon comparison of neonates of insulin and metformin treated GDMs. He also has found non-significant results for Apgar score at 5 minutes.^15^ In our study females who received insulin delivered late as compared to females on metformin therapy and the results were statistically significant (p=0.021). Kelley stated that there was significantly less maternal weight gain and lower gestational age at delivery with metformin compared with insulin therapy.^16^ Whereas Territi stated that there was no difference in both the groups when delivery at gestational age was tabulated.^17^ There were two intrauterine deaths in insulin groups in our study whereas all the babies were delivered healthy in metformin group confirms that metformin is a safer and effective drug. Rowan stated that 0.2% of females had intra uterine deaths with insulin whereas none of the patient in metformin group had such an issue.^15^ However results from Hellmuth provide contradictory results to ours, as more intrauterine deaths were observed by him in metformin treated group then in insulin treated group.^18^ The probable cause could be that females in his study on metformin started treatment in last trimester, when already a level of irreversible hypoxic changes in placenta and fetus had taken place. Carson stated that insulin infusions for several days to the fetal lamb produce a progressive decline in fetal arterial oxygen content and may be one of the factors for more IUDs with insulin treatment.^19^

With regard to maternal outcomes, it was observed that mean fasting blood sugar at 36 weeks was lowered numerically and HbA_I_C was significantly less in metformin treated patients as compared to insulin treated GDMs. So we can say that metformin has fulfilled the demands and needs of an anti-glycemic agent. Results by Riaz are in agreement with ours as he documented that metformin for the glycemic control in gestational diabetes mellitus is significantly superior to insulin.^20^ With regard to mode of delivery, it is clear from our results that surgical deliveries due to Feto-maternal disproportion were in insulin treated patients than in metformin treated patients with a ratio of 7: 2 respectively. These two females in metformin group had surgical deliveries due to narrow pelvic outlet though the babies even in these two cases were not large in size. Goh has highlighted that the caesarean section rate in women with insulin treated GDM was 45.6% due to Feto-maternal disproportion which is much higher than metformin treated group.^21^ Kale also stated that patients on insulin therapy for GDM have higher rates for cesarean sections and his results are similar to ours.^22^ Moore discovered that metformin is an effective alternative to insulin as there was no significant difference present in mode of delivery, fetal weight and neonatal complications between insulin treated and metformin treated female in GDMs which is contradictory to our results.^23^ Waheed also reported that no significant differences were present in mode of delivery and fetal complications, when patients on insulin and metformin treatment for gestational diabetes were compared.^3^

We also compared the results in both the groups for glycemic values, at the time of enrollment and after the treatment at 36 weeks to evaluate the effectiveness of the treatment. The results showed that metformin decreases the fasting blood glucose levels before and after the treatment significantly (p=0.00). HbA_I_C was non-significant when compared between the start of study and at term (p=0.061) clearly indicating that metformin has maintained the sugar levels and there was no predicted rise with the advancement of pregnancy. Whereas insulin group showed significant increase in fasting blood sugar levels as well for HbA_I_C when compared before and after the treatment, (FBS, p=0.002 &HbA_I_C, p=0.04) indicating that insulin was unable to show expected results. Mesdaghinia declared similar results that not only end term HbA_I_C but also maternal and fetal complications were much higher in Insulin group and metformin showed better glycemic control and feto-maternal outcome.^24^ A recent study by Saleh also confirmed the fact that good glycemic control can be achieved using metformin with better fetal and maternal outcomes.^25^

## CONCLUSION

Metformin has produced better effects on feto-maternal outcomes and glycemic control in patients with gestational diabetes in comparison to insulin. Metformin is used through physiological route, has no storage problem and nor does it requires any dependency. With all these features metformin is a better option than insulin in our population for patients having gestational diabetes mellitus. However multi-centric studies with large sample size are open venues for future research.

### Source of Funding

Self and Dow University of Health Sciences.

### Work Place

Dow Diagnostic and Research Laboratory, Dow University of Health Sciences, Karachi, Pakistan.

### Authors` Contribution

**RA** perceived, designed, conducted research and did main write up of manuscript.

**SK** helped in pathological aspects, write up and editing of manuscript.

**FS** helped in pharmacological aspects, data collection and proof reading of manuscript.

**NK** revised it critically for important intellectual content and is supervisor of the study.
